# Oral Microbiota Is Associated With Immune Recovery in Human Immunodeficiency Virus-Infected Individuals

**DOI:** 10.3389/fmicb.2021.794746

**Published:** 2021-12-09

**Authors:** Yirui Xie, Jia Sun, Caiqin Hu, Bing Ruan, Biao Zhu

**Affiliations:** ^1^State Key Laboratory for Diagnosis and Treatment of Infectious Diseases, Department of Infectious Diseases, National Clinical Research Center for Infectious Diseases, Collaborative Innovation Center for Diagnosis and Treatment of Infectious Diseases, School of Medicine, The First Affiliated Hospital, Zhejiang University, Hangzhou, China; ^2^Ningbo Medical Center Lihuili Hospital, Ningbo, China

**Keywords:** HIV-1, oral microbiota, immunological responders, immunological non-responders, antiretroviral therapy

## Abstract

The role of the oral microbiota in HIV-infected individuals deserves attention as either HIV infection or antiretroviral therapy (ART) may have effect on the diversity and the composition of the oral microbiome. However, few studies have addressed the oral microbiota and its interplay with different immune responses to ART in HIV-infected individuals. Salivary microbiota and immune activation were studied in 30 HIV-infected immunological responders (IR) and 34 immunological non-responders (INR) (≥500 and < 200 CD4 + T-cell counts/μl after 2 years of HIV-1 viral suppression, respectively) with no comorbidities. Metagenome sequencing revealed that the IR and the INR group presented similar salivary bacterial richness and diversity. The INR group presented a significantly higher abundance of genus *Selenomonas_4*, while the IR group manifested higher abundances of *Candidatus_Saccharimonas* and *norank_p_Saccharimonas. Candidatus_Saccharimonas* and *norank_p_Saccharimonas* were positively correlated with the current CD4 + T-cells. *Candidatus_Saccharimonas* was positively correlated with the markers of adaptive immunity CD4 + CD57 + T-cells, while negative correlation was found between *norank _p_Saccharimonas* and the CD8 + CD38 + T-cells as well as the CD4/CD8 + HLADR + CD38 + T-cells. The conclusions are that the overall salivary microbiota structure was similar in the immunological responders and immunological non-responders, while there were some taxonomic differences in the salivary bacterial composition. *Selenomona_4, Candidatus_Saccharimonas*, and *norank _p_Saccharimonas* might act as important factors of the immune recovery in the immunodeficiency patients, and *Candidatus_Saccharimonas* could be considered in the future as screening biomarkers for the immune responses in the HIV-infected individuals.

## Introduction

The widespread use of potent antiretroviral therapy (ART) which has ability to achieve viral suppression and immune reconstitution has made human immunodeficiency virus (HIV) infection become a chronic manageable disease. ART can improve the immune function of HIV infected subjects, but significant individual difference exists in the extent of immunological recovery. Despite the persistent manifestation of virological suppression after receiving ART, HIV infected individuals with low increases of CD4 + T-cells are considered as immunological non-responders (INR), showing contrast to immunological responders (IR) (Cenderello and De Maria, 2016). An unanimously agreed definition of INR has not been reached by far. Therefore, the acceptable range of the prevalence of INR is from 10 to 40% in the cohorts (Yang et al., 2020). The most restrictive definition of INR refers to patients whose absolute CD4 + T-cells fail to reach 200 cells/μl with an undetectable plasma viral load after 2 years of receiving ART (Florence et al., 2003; Kaufmann et al., 2003; Tsukamoto et al., 2009).

Thanks to high-throughput sequencing, thorough and comprehensive studies of complex human microbiology have been conducted during the past years. In recent years, studies mainly focused on characterizing the impact of HIV infection on host–microbe interactions in the gut (Dillon et al., 2016) and some studies found that the gut microbiota are associated with immune recovery in HIV-infected patients (Ji et al., 2018; Lu et al., 2018; Xie et al., 2021). Oral microbiomes were also reported to have strong associations with human immune system functions, therefore, they were correlated with human immune system diseases such as rheumatic diseases (RDs), acute lymphoblastic leukemia (ALL), and HIV infection (Gao et al., 2018). Alterations in the oral microbiome distinguished individuals with rheumatoid arthritis (RA) from the healthy controls; correlations were shown between these alterations and clinical measures including immune response, and they could be used to stratify individuals based on their responses to the therapy, especially with microbial triggers being implicated in RA (Bellando-Randone et al., 2021). Furthermore, characterization of the oral microbiome in ALL patients demonstrated a structural imbalance of the oral microbiota, indicating the importance of immune status in shaping the structure of the oral microbiota. Although valuable insights on immune status have been presented by these studies, it is surprising that studies which focus on the role of the oral microbiome and their relationship with immune response in HIV are relatively rare. Few research addresses the overall oral microbiota structure in HIV infection; to our knowledge, the oral microbiota dysbiosis and the interaction with different immune responses to ART is still poorly defined. This study aims to evaluate the microbial composition in the salivary samples collected from HIV-infected immunological non-responders and immunological responders. We hypothesize that the compositional changes of the salivary microbiota could be associated with different immune responses of HIV-infected individuals receiving ART. The study adopted 16S ribosomal RNA (rRNA) targeted sequencing and flow cytometry to explore the oral microbiome and their relationship with immune activation in patients who are immunodiscordant and who are immunoconcordant. The compositional changes of salivary microbiota and their association with different immune responses in HIV-infected patients with ART is characterized for the first time in this study.

## Materials and Methods

### Recruitment of Subjects

64 HIV-infected individuals who were diagnosed by the Disease Control and Prevention Center of Zhejiang Province (30 immunological responders and 34 immunological non-responders) were all recruited from the HIV clinic of the First Affiliated Hospital of Zhejiang University from November 2015 to October 2017. All subjects start ART during the chronic phase of HIV infection. In this study, IR and INR were defined as patients who with the average of the last two CD4 + T-cell counts/μl equal or is more than 500 or less than 200 and after 2 years of receiving complete viral suppression therapy, respectively. The selection excluded candidates with either one of the following conditions: below 18 years old; showing opportunistic infection symptoms; infected with hepatitis B or C; having history of using antibiotics, immunosuppressive regimen, probiotics, prebiotics, or symbiotics in the past 6 months; BMI higher than 30; showing oral active inflammation. None of the patients had obvious symptoms of oral mucosal diseases and periodontal disease (redness, swelling, and bleeding) when the clinical samples were collected. However, formal dental examinations were not performed to rule out the mild periodontal symptoms.

### Ethics Statement

This study conforms to the ethical norms of the 1975 Helsinki Declaration. The research protocol was approved by the Institutional Review Committee of The First Affiliated Hospital of Zhejiang University on October 7, 2015. All participants provided written informed consents before participating in the study. All the data used for analysis were anonymized.

### Salivary Samples Collection and DNA Extraction

Participants were required to refrain from eating, drinking, smoking before saliva collection. The amount of each salivary sample collected from participants before their clinic visit was 5 ml. The samples were stored in sterile containers of –80°C until DNA extraction by QiaAmp DNA Mini Kit (QIAGEN, Hilden, Germany) following instructions of the manufacturer. NanoDrop (Thermo Fisher Scientific) was used to determine the concentration and purity of DNA as well as 1.0% agarose gel electrophoresis for the integrity and size of DNA. After the procedures above, the DNA samples were frozen at –20°C for further analysis.

### 16S Ribosomal RNA Gene Sequencing

The bacterial 16S rRNA gene high-throughput sequencing was conducted by Shanghai Majorbio Bio-Pharm Technology Co., Ltd. (Shanghai, China). The bacterial 16S rRNA gene sequences spanning the variable regions V3–V4 were amplified using the primer 338F (5′- ACTCCTACGGGAGGCAGCAG-3′), and 806R (5′-GGACTACHVGGGTWTCTAAT-3′) as previous recorded (Xie et al., 2021). The amplicons were extracted from 2% agarose gels, purified by the AxyPrep DNA Gel Extraction Kit (Axygen Biosciences, Union City, CA, United States) and quantified using QuantiFluor™-ST (Promega, United States) based on the guidelines of the manufacturer’s protocol. In equimolar amounts, purified amplicons were sent to paired-end sequencing (2 × 300) on an Illumina MiSeq platform (Illumina, San Diego, United States).

### Bacterial Translocation, Viral Load, and Flow Cytometry

Sera samples of immunological responders and non-responders were collected to measure the bacterial translocation markers. Following the standard protocols, human Lipopolysaccharides (LPS) ELISA Kit (CUSABIO; Wuhan, China) and Human soluble CD14 (sCD14) ELISA Kit (MultiSciences, Hangzhou, China) were used to test plasma LPS and sCD14. Flow cytometry and Cobas Amplicor (Roche Molecular Systems Inc., Branchburg, New Jersey, United States) were used to quantify CD4 + /CD8 + T-cells and HIV-1 RNA, respectively. Fresh anticoagulated whole blood was used to quantify the expressing markers of immune activation (CD25 +, CD38 +, HLADR +, or CD38 + /HLA-DR +) of CD4 + and CD8 + T-cells and immune senescence (CD57 +) by BD FACS Canto II flow cytometer (BD Biosciences, California, United States). The antibodies needed during the experiment were purchased from Biolegend (San Diego, CA), including CD3-FITC, CD4- PerCP/Cy5.5, CD8-Brilliant Violet 510™, CD38-Brilliant Violet 421, CD25-PE, HLA-DR-APC/Fire™ 750, and CD57-allophycocyanin (APC).

### Bioinformatics and Statistics

The 16S rRNA high-throughput sequencing raw fastq files were demultiplexed and quality-filtered by QIIME (version 1.9.1).^1^ Operational taxonomic units (OTUs) were clustered with a 97% similarity cut-off using UPARSE.^2^ The taxonomy of each 16S rRNA gene sequence was analyzed using the RDP Classifier (version 2.2)^3^ and compared with the SILVA rRNA database^4^ with the confidence threshold being 70%. After eliminating the interference sequence, alpha diversity estimator calculations were performed using Mothur v.1.30.2. Phylogenetic beta diversity measures, such as Bray-Curtis distance metrics analysis; the representative sequences of OTUs were used for each sample, respectively. Principal Coordinates analysis (PCoA) was performed to visualize the microbial communities following the distance matrices, as well as Linear discriminant analysis effect size (LEfSe) on Galaxy to calculate bacteria taxa with significantly different abundances between groups (Segata et al., 2011). In this study, alpha values for the factorial Kruskal Wallis at 0.05 and linear discriminant analysis (LDA) effect size threshold of 2.0 for discriminative features were applied for all bacteria that were discussed. The calculation of correlations between the variables were conducted with Spearman’s rank-correlation analysis. Spearman correlation matrix with *p*-adjust lower than 0.05 and ρ-value above 0.2 were used to filter strong correlations. The discriminatory function of the biomarkers was evaluated through calculating the area under the receiver operating characteristic (ROC) curve (AUC) using pROC of R package. The comparisons between groups were conducted through the Chi-square test, Independent-Samples *T*-test, Wilcoxon rank sum test and Mann-Whitney *U*-test in the R package and SPSS 21.0 software (SPSS Inc., Chicago, IL, United States). Differences were considered significant when *P* < 0.05.

## Results

### General Clinical Features of the Patients

The cross-sectional study subjects included 30 immunological responders and 34 immunological non-responders with HIV infection. The characteristics such as gender, age, body mass index (BMI), and smoking status are relatively matched between the two groups (Table 1). The MSM transmission route rate is 53.3% vs. 44.1% (*p* = 0.461).

**TABLE 1:** Clinical characteristics data summary.

Characteristics	IR (*n* = 30)	INR (*n* = 34)	*P-*value
Age, (years), mean ± SD	36.97 ± 9.91	38.59 ± 9.45	0.506
Gender male/female	27/3	33/1	0.224
BMI, mean ± SD	21.31 ± 2.60	20.67 ± 2.58	0.368
Mode of transmission, MSM, N (%)	16 (53.3%)	15 (44.1%)	0.461
Smoking, N (%)	0 (0%)	1 (2.94%)	0.096
HAART months (mean ± SD)	37.60 ± 13.21	33.61 ± 10.20	0.193
**Ongoing ART regimen, N (%)**			
NNRTI-based PI-based	27 (90.0%) 3 (10.0%)	30 (88.2%) 4 (11.8%)	0.821 0.821
Nadir CD4 + T cell count, /mm^3^, median (IQR)	298 (200, 371)	42.5 (13, 140.3)	**<0.001**

*Continuous variables were compared using Independent-Samples t-test or the Mann-Whitney U-test. Categorical variables were compared using Chi-square test or Fisher’s exact test. BMI, body mass index; MSM, men who sex with men; NNRTI, Non-nucleoside reverse transcriptase inhibitors; PI, Protease inhibitor; IR, immunological responders; INR, immunological non-responders. Bold values indicate P < 0.05.*

ART medications were composed by two Nucleoside/nucleotide reverse transcriptase inhibitors (NRTIs) and a non-nucleoside reverse transcriptase inhibitor (NNRTI), or a ritonavir-boosted protease inhibitor (PI). No differences are observed in the duration of ART and the type of ART drugs between the IR and the INR groups (*p* = 0.193 and *p* = 0.821). The HIV RNA viral load levels were considered as undetectable (<20 copies/ml) in all samples of the patients. In the IR group, the number of Nadir CD4 + T cell is greatly higher than that in the INR group (298 vs. 42.5, *p* < 0.001) (Table 1).

### Salivary Microbiota Analysis in the Immunological Responders and Immunological Non-responders Groups

**FIGURE 1:**
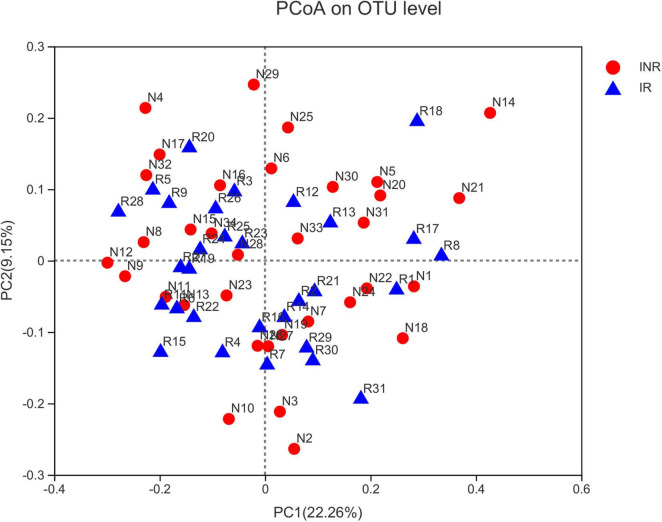
Principal coordinates analysis (PCoA) of salivary microbiota in the IR and INR groups. No significant difference of bacterial communities between the immunological responders (IR) and immunological non-responders (INR) groups. IR, immunological responders; INR, immunological non-responders.

**FIGURE 2:**
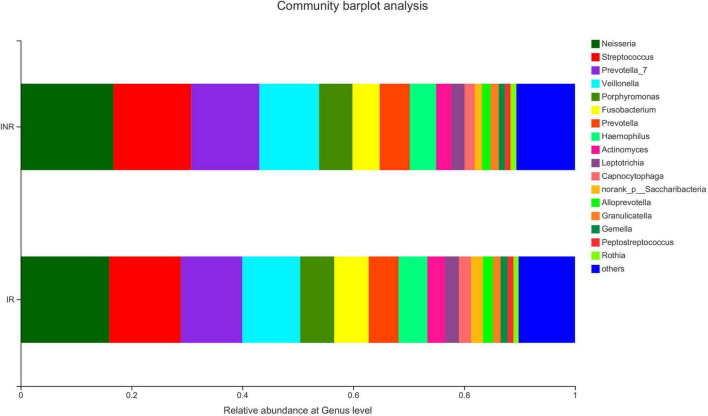
The salivary microbiota composition at genus level in the IR and INR groups. IR, immunological responders; INR, immunological non-responders.

**FIGURE 3:**
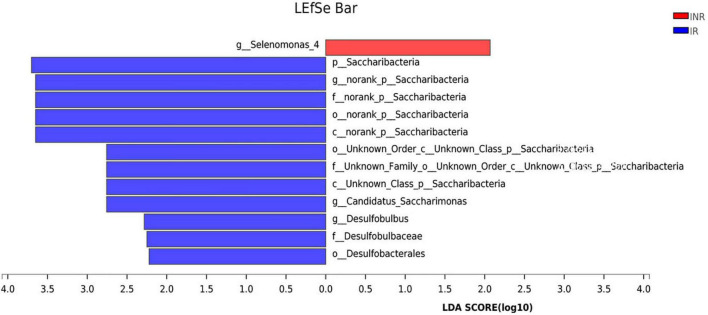
Linear discriminative analysis (LDA) effect size (LEfSe) analysis in the IR and INR groups. LDA scores for the significant taxa in the immunological non-responders (INR) group are represented on the positive scale (red), and LDA-negative scores represent enriched taxa in the immunological responders (IR) group (blue). IR, immunological responders; INR, immunological non-responders.

**FIGURE 4:**
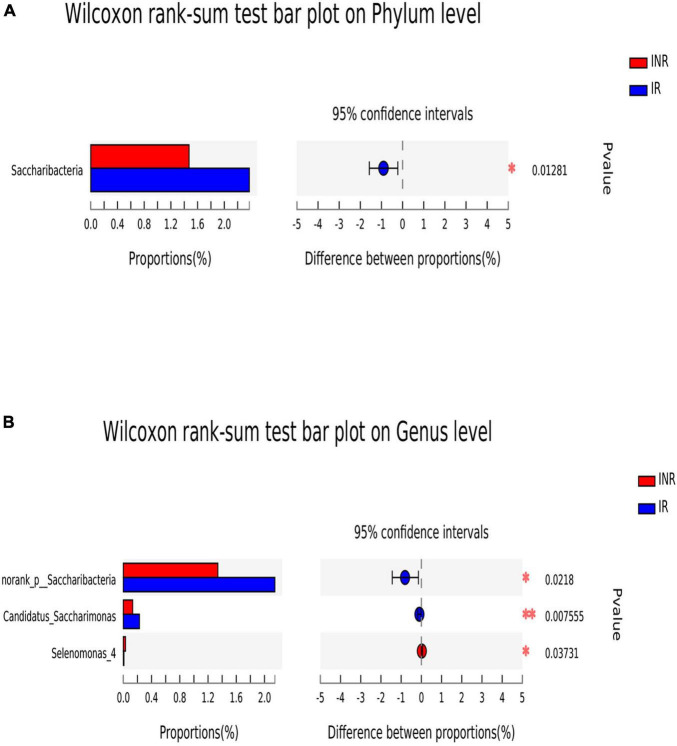
The difference of saliva microbial taxa between the IR and INR groups at the phylum and genus levels. The Wilcoxon Rank Sum test was performed to detect taxa with significant differences in relative abundances at the phylum **(A)** and the genus levels **(B)** between the two groups (using confidence interval method). IR, immunological responders; INR, immunological non-responders. *P < 0.05 and ^**^P < 0.01.

To characterize the salivary microbiota composition, 3,884,775 high-quality16S rRNA sequences were obtained from all 64 participants. An average length of 445 bp and an average of 60,700 sequences per sample were adopted for further analysis. Rarefaction was conducted on the OTU table to 27,986 reads per sample to avoid any methodological artifacts. Specifically, 504 OTUs in the IR group and 503 OTUs in the INR group were defined at a similarity level of 97%. The diversity (Sobs index, Simpson’s index of diversity and Shannon index) and richness (Chao1, ACE estimator and Good’s coverage) of the salivary microbiota in each group at the OTU level were analyzed, and the IR group present similar Alpha and Beta diversity compared to the INR group (Table 2). It can be seen from the principal coordinate (PCoA) analysis by Bray-Curtis matrices that there is no significant difference between the two groups (PERMANOVA, pseudo-F: 1.64608, *R*^2^ = 0.02586, *p* = 0.109, Figure 1). Visualization of the relative abundances of dominant taxa at the genus level in the oral microbiome is presented in Figure 2. In order to identify the key phylotypes responsible for the difference in distinguishing the saliva microbiota between the two groups, linear discriminant analysis (LDA) effect size (LEfSe) was performed and the threshold was 2. The INR group present a significantly higher abundance of genus *Selenomonas_4*, while the IR group has higher abundances of genus *Candidatus_Saccharimonas, norank _p_Saccharimonas*, and *Desulfobulbus* (Figure 3). The Wilcoxon Rank Sum test was also used to detect the taxa with significant differences in the relative abundances between the two groups (using confidence interval method). The abundance of *Saccharimonas* is more abundant in the IR group than the INR group at the phylum level (Figure 4A). Compared with the INR group, the abundances of genus *Candidatus_Saccharimonas* and *norank_p_Saccharimonas* are dramatically increased in the IR group, while the abundance of genus *Selenomonas_4* is dramatically decreased in the IR group (Figure 4B).

**TABLE 2:** Salivary microbiota 16S rRNA gene high-throughput sequencing data summary.

Characteristics	IR (*n* = 30)	INR (*n* = 34)	*P-*value
Sobs index^#^	268.93 ± 45.29	262.79 ± 40.93	0.716
Shannon index^#^	3.56 ± 0.27	3.50 ± 0.29	0.471
Simpson index^#^	0.06 ± 0.02	0.07 ± 0.02	0.245
ACE^#^	306.14 ± 49.96	295.87 ± 44.66	0.497
Chao 1 index^#^	310.13 ± 54.65	301.49 ± 48.64	0.568
Good’s coverage (%)^#^	99.85 ± 0.04	99.84 ± 0.03	0.404

*^#^Indicate the diversity and richness was calculated after the reads number of each sample were equalized. IR: immunological responders; INR: immunological non-responders.*

### Comparison of Adaptive Immunity and Bacterial Translocation Markers in the Immunological Responders and Immunological Non-responders Groups

As expected, the amount of the current CD4 + T-cell counts and the CD4/CD8 ratio in the INR group is lower than those in the IR group (*p* < 0.001). The proportion of the CD8 + CD38 + T-cell and the CD8 + CD57 + T-cell is significantly higher in the INR group than those in the IR group (*p* = 0.032 and *p* = 0.001). The proportion level of the CD4 + immune activation (CD4 + T-cell by the expression of CD25 +, HLA-DR +, CD38 +, or HLA-DR + /CD38 +) shows similarity in the INR and IR groups. Lipopolysaccharide (LPS), which is commonly used as the major antigens driving chronic immune activation, is significantly higher in the INR group compared with that in the IR group (*p* = 0.027) (Table 3).

**TABLE 3:** Comparison of adaptive immunity and bacterial translocation markers.

Characteristics	IR (*n* = 30)	INR (*n* = 34)	*P-*value
Current CD4 + T cell count,/mm^3^, median (IQR)	597 (466.5, 666.5)	209 (163.3, 303)	**<0.001**
Current CD4 + /CD8 + T-cell ratio	0.9 (0.5, 1)	0.4 (0.2, 0.5)	**<0.001**
%CD4 + HLADR + CD38 +	7.2 (4.5, 10)	7.8 (4.7, 12.4)	0.418
%CD4 + CD25 +	1.1 (0.6, 1.5)	0.8 (0.3, 1.6)	0.388
%CD4 + CD57 +	2.3 (0.7, 4.1)	1.1 (0.2, 2.9)	0.083
%CD8 + CD38 +	31.6 (25.7, 39.2)	44.1 (28.0, 58.0)	**0.032**
%CD8 + HLADR + CD38 +	17.8 (13.1, 26.5)	23.7 (12.4, 35.7)	0.388
%CD8 + CD57 +	13.4 (10.5, 19.8)	21.7 (14.2, 34.5)	**0.001**
LPS (pg/ml, mean ± SD)	64.7 (50.6, 104.4)	90.1 (65.5, 152)	**0.027**
sCD14 (pg/ml, mean ± SD)	2052.1 (1797.7, 2413.5)	2319.8 (1878.1, 2654.4)	0.169

*IR, immunological responders; INR, immunological non-responders. Bold values indicate P < 0.05.*

### Associations Between Oral Microbiome and Adaptive Immunity

**FIGURE 5:**
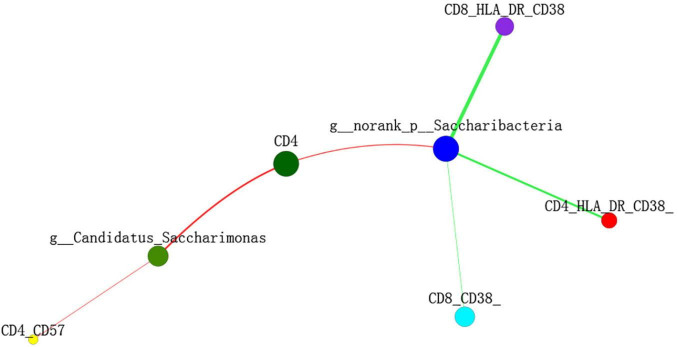
Two-way correlation network analysis between the saliva microbiota and the adaptive immunity markers. Some adaptive immunity markers are correlated with the specific genera of saliva microbiota. Positive correlation is shown in the red line, negative correlation is shown in the green line. Spearman’s correlation was used, and associations with *p*-adjust lower than 0.05 and ρ-value above 0.2 were considered relevant.

**FIGURE 6:**
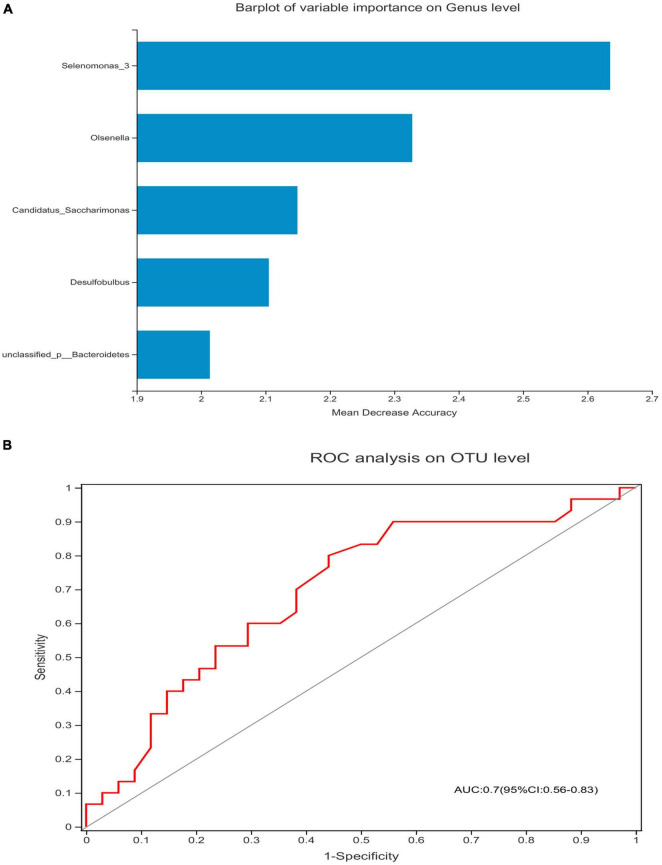
*Candidatus_Saccharimonas* could be used to discriminate the IR from the INR group. Barplot of variable importance on the genus level **(A)** and the receiving operational curve analysis were performed **(B)** [area under curve (AUC) = 0.7]. Diagonal lines represent random classifications (AUC = 0.5).

The Spearman’s correlation test was used to investigate the correlation between the relative abundance of the different genera and adaptive immunity markers. The *p*-value was corrected by FDR, *p*-adjust lower than 0.05 with ρ-value above 0.2 were considered relevant and showed in Figure 5. As the abundance of genus *Selenomonas_4* is low in the two groups, the multiple correlation analyses were not performed. *Candidatus_Saccharimonas* and *norank _p_Saccharimonas* are positively correlated with the current CD4 + T cells (*p*-adjust = 0.033 and 0.025, respectively). *Candidatus_Saccharimonas* is positively correlated with the markers of the adaptive immunity CD4 + CD57 + T-cells (*p*-adjust = 0.049), while *norank _p_Saccharimonas* is negatively correlated with the CD8 + CD38 + T-cells and the CD4/CD8 + HLADR + CD38 + T-cells, respectively (*p*-adjust = 0.001 and 0.032, respectively) (Figure 5). To explore the potential function of the saliva microbiome for discriminating the IR and INR status, a random forest model was created based on the microbiome and the top 5 genus was shown in Figure 6A. The ROC analysis shows that *Candidatus_Saccharimonas* could be used to discriminate the IR from the INR group [ROC-plot AUC value of 0.7 (95% CI, 0.56–0.83), Figure 6B].

## Discussion

It is well-known that there is a dynamic interaction between the host and the microbiota, which is a major factor of people’s health (Dethlefsen et al., 2007). Current available studies have confirmed that discernible alterations of the composition of the salivary microbiota are inherent to a range of systemic disorders. Studies on oral microbiomes of human immune system diseases such as RA, ALL, and HIV have also indicated that the oral microbiota is strongly related to the immune responses in immunodeficiency patients (Acharya et al., 2017; Corrêa et al., 2017; Annavajhala et al., 2020; Bellando-Randone et al., 2021). Furthermore, the hypothesis that oral microbiota is associated with different immune responses to ART is supported by the high frequency of opportunistic oral infections in HIV-infected patients and its association with CD4 + T cells levels (Berberi and Noujeim, 2015). As stated, the role of the oral microbiota played in HIV-infected patients deserves much attention because the diversity and composition of the oral microbiome can be changed by HIV infection or by ART (Heron and Elahi, 2017); however, the results are highly variable. Previous studies have observed the generally similar structure of salivary microbiota as well as the differences in the relative abundances of several bacterial taxa between HIV-infected subjects and uninfected controls (Li et al., 2014; Kistler et al., 2015; Mukherjee et al., 2018; Lewy et al., 2019). However, significant differences were also found by several studies in the saliva bacterial communities between HIV-infected and uninfected individuals (Hegde et al., 2014; Mukherjee et al., 2014; Beck et al., 2015; Goldberg et al., 2015; Kistler et al., 2015; Li et al., 2021). What’s more, some studies reported significant distinctions in the prevalence and the distribution of the saliva bacterial communities among HIV-infected individuals before and after the antiretroviral therapy (Li et al., 2014; Kistler et al., 2015; Mukherjee et al., 2018). Few studies have addressed the interaction of oral microbiota with different immune responses to ART received by the HIV-infected individuals. In this paper, we demonstrate the oral microbiota structure and its relationship with immune response in HIV infection.

A previous study indicated that after receiving 24 weeks of ART, the salivary microbiome in the three HIV-infected participants with persistently low CD4 + T-cell count had significantly higher bacterial richness and Shannon diversity; when compared to those with CD4 counts that remained or recovered to greater than 200 cells/μl. Several taxa with different abundancies, such as *Porphyromonas* species, discriminated between the baseline and the posttreatment samples; this suggested that the salivary microbiome can be an important factor in the CD4 + T-cell count recovery after ART in the study (Presti et al., 2018). However, the major limitation of this study is the insufficient number of studied subjects and the short time period of patients receiving ART. Thus, more studies on larger cohorts are necessary for a better understanding of the potential roles of different immune responses to ART on oral microbiome. Making the most use of the non-invasive and unsuspicious functions of saliva sampling, we collected the salivary samples from the HIV-infected immunological non-responders and immunological responders and studied the salivary microbiome using high-throughput sequencing technology. To our knowledge, this is the first study that has fully utilized the next-generation sequencing technology to characterize and compare the community composition of the salivary microbiota in the higher number of HIV-infected immunological responders and non-responders. These findings are particularly important given that the adaptive immunity markers assessments were performed on all participants, offering an opportunity to evaluate the relationship between the salivary microbiota and the immunologic markers in the immunological responders and non-responders.

The IR group presented similar salivary bacterial richness and diversity when compared with the INR group in this study. A similar Alpha diversity of salivary bacterial community was also described in the HIV-infected IR (*n* = 18) and INR (*n* = 9) individuals in a previous study (Jiménez-Hernández et al., 2019). While another study reported that the three HIV-infected participants with persistently low CD4 + T-cell counts had significantly higher salivary bacterial richness and Shannon diversity after their 24 weeks of ART, when compared with the participants whose CD4 + T-cell counts higher than 200 cells/μl (Presti et al., 2018). It claimed that the HIV infection and highly active antiretroviral therapy (HAART) had significant effects on salivary microbial colonization and composition, and *Selenomonas* noticeably increased after HAART (Li et al., 2014), which were reported to be depleted in the oral microbiome of the HIV-associated periodontitis (Noguera-Julian et al., 2017). For patients with systemic lupus erythematosus (SLE), increased numbers of *Selenomonas* were directly correlated with the elevated levels of inflammatory cytokines IL-6, IL-17, and IL-33 (Corrêa et al., 2017). In this study, when we visualized the relative abundances of dominant taxa at the genus level in the oral microbiome, we found that the INR group presented a significantly higher abundance of genus *Selenomonas_4.* Gut *Candidatus_Saccharimonas* was reported decreased in rats acute necrotizing pancreatitis (Chen et al., 2017), which indicated that *Candidatus_Saccharimonas* did play an important role in maintaining normal intestinal functions. A previously published study stated that the relative abundance of gut *Candidatus_Saccharimonas* was negatively correlated with the expression levels of cadherin-11, IL-17α, and TLR2 in the adjuvant-induced arthritis rat model (Huang et al., 2019). In this study, the IR group had higher abundances of genus *Candidatus_Saccharimonas* and *norank _p_Saccharimonas* when compared with the INR group. In addition, *Candidatus_Saccharimonas* had positive correlation with the CD4 + T-cells and CD4 + CD57 + T-cells, while *norank _p_Saccharimonas* was positively correlated with the CD4 + T-cells but negatively correlated with the CD8 + CD38 + T-cells and CD4/CD8 + HLADR + CD38 + T-cells. While host biomarkers were subjected to the individual biological variations, oral microbiome was relatively conserved among unrelated individuals. Recent advances of saliva analysis have played a key role in the definitions of biomarkers for the diagnosis, prognosis, and the treatment of human immune system diseases (Bellando-Randone et al., 2021). Expansion of specific microbial consortia in the saliva may act as imprints of the underlying immuno-inflammatory processes, especially in HIV. This study indicated that *Selenomona, Candidatus_Saccharimonas*, and *norank _p_Saccharimonas* might all played important roles in the immune recovery of the immunodeficiency patients, and *Candidatus_Saccharimonas* could be considered in the future as screening biomarkers for immune responses in HIV-infected individuals, leading to the future design of effective individualized treatment strategies such as probiotics for the immunological non-responders. However, a previous study on the effect of the prebiotic modulation of the salivary microbiota in HIV-infected patients with diverse immunopathogenesis stated that: *Streptococcus anginosus* was in correlation with the CD4 + T cells, *Veillonella parvula* with the CD4 + CD25 + T cells, and *Prevotella pallens, Prevotella copri, and Prevotella nigrencens* with the markers of adaptive immunity such as CD4 + CD25 + T cells, CD4 + CD57 + T cells, and CD4 + HLADR + CD38 + T cells, respectively (Jiménez-Hernández et al., 2019). The inconsistent results may be caused by the different number of subjects in the two studies and their different definitions of INR. More studies need to be carried out to determine the precise cause of this inconsistency.

This study provides a better understanding of the oral microbial profiles and their relationship with the immune responses in the immunocompromised patients. Although the correlations between the salivary microbiota and the biomarkers of adaptive immunity were observed, we could not establish a causal influence between the oral microbiome and the immune system in the HIV-infected patients. Thus, further research on the exact mechanisms involved in the interaction between the immune system and oral microbiota is still required. We find the low number of participants, absence of control group, and cross-sectional analysis caused limitations to the explanation of our findings. Intra-person variability will need larger longitudinal studies with control group, which should involve plaque collection and the observation of the clinical and environmental changes that account for intra-person variability over time. Apart from taxonomic characterization, there should be more studies on identifying bacterial pathways and the resulting metabolites that promote disease and immunity.

In summary, this study focuses on the overall oral microbiota structure and its interactions with different immune responses to ART. While there were some taxonomic differences in the salivary bacterial composition, the results suggested that the overall structure of the salivary microbiota in the immunological responders was similar with those that were in the immunological non-responders. *Selenomona_4, Candidatus_Saccharimonas*, and *norank_p_Saccharimonas* might act as important factors of the immune recovery in the immunodeficiency patients, and *Candidatus_Saccharimonas* could be considered in the future as screening biomarkers for immune responses in the HIV-infected individuals.

## Data Availability Statement

Publicly available datasets were analyzed in this study. This data can be found here: The raw reads were deposited into the NCBI Sequence Read Archive (SRA) database and is accessible with the following link: https://dataview.ncbi.nlm.nih.gov/object/PRJNA744583?reviewer=3gmrc9a67n88rhe2gr9ehub4ok.

## Ethics Statement

The research protocol was approved by the Institutional Review Committee of The First Affiliated Hospital of Zhejiang University on October 7, 2015. The patients/participants provided their written informed consent to participate in this study.

## Author Contributions

YX participated in the designing of the study and wrote the manuscript. JS performed the statistical analysis and revised the manuscript. JS and CH collected the biopsy samples and carried out the experiment. BR and BZ participated in the designing and reviewing of the manuscript. All authors read through and approved the final manuscript.

## Conflict of Interest

The authors declare that the research was conducted in the absence of any commercial or financial relationships that could be construed as a potential conflict of interest.

## Publisher’s Note

All claims expressed in this article are solely those of the authors and do not necessarily represent those of their affiliated organizations, or those of the publisher, the editors and the reviewers. Any product that may be evaluated in this article, or claim that may be made by its manufacturer, is not guaranteed or endorsed by the publisher.

## Footnotes

1
http://qiime.org/install/index.html

2
http://drive5.com/uparse/

3
http://rdp.cme.msu.edu/

4
http://www.arb-silva.de

## Abbreviations

HIV: human immunodeficiency virus
ART: antiretroviral therapy
HAART: highly active antiretroviral therapy
INR: immunological non-responders
IR: immunological responders
SLE: systemic lupus erythematosus
RDs: rheumatic diseases
ALL: acute lymphoblastic leukemia
RA: rheumatoid arthritis
BMI: body mass index
MSM: men who have sex with men
LPS: Lipopolysaccharide
PCoA: Principal coordinate analysis
LDA: Linear discriminant analysis
LEfSe: linear discriminant analysis effect size
OUT: operational taxonomic unit
16S rRNA: 16S ribosomal RNA
ROC: receiver operating characteristic
NRTIs: nucleoside reverse transcriptase inhibitors
NNRTIs: non-nucleoside reverse transcriptase inhibitors
AZT: Zidovudine
TDF: Tenofovir Disoproxil Fumarate
3TC: Lamivudine
EFV: Efavirenz
LPV/r: Lopinavir/ritonavir.
